# High and Rapid Uptake of COVID-19 Vaccine Among Chicago Women with and Without HIV

**DOI:** 10.1089/whr.2024.0197

**Published:** 2025-04-21

**Authors:** Elizabeth Daubert, Mardge H. Cohen, Tsion Yohannes, Darlene Johnson, Calvine Thompson, Andrea C. Rogando, Ralph Morack, Audrey L. French, Kathleen M. Weber

**Affiliations:** ^1^ Hektoen Institute of Medicine, Chicago, Illinois, USA.; ^2^ Department of Medicine, Stroger Hospital of Cook County, Chicago, Illinois, USA.

**Keywords:** COVID-19, vaccine intent, vaccine uptake, vaccine refusal, HIV, women

## Abstract

**Background:**

Chicago sustained substantial COVID-19 morbidity and mortality with greatest burdens among low-income communities of color. We sought to determine the prevalence and predictors of vaccine uptake and refusal over 3 years among a long-term cohort of Chicago women with/without HIV (WWH/WWoH).

**Methods:**

Research staff provided outreach and collected data on COVID-19 knowledge, vaccine intent, uptake, and refusal quarterly during 2020 and then semiannually through 2023. 146 women (102 WWH and 44 WWoH) participated.

**Results:**

Among 146 women, mean age was 54.4 years, 70% were WWH; predominantly Black (63%), unemployed (73%), 58% had ≤$18,000 annual household income, 63% had a high school education or less, and 65% had one or more comorbidities. Initially, 46% of women reported no intent to be vaccinated and were more likely to be employed, report medical mistrust and not living with HIV. By September 2023, 88% of women received at least one vaccination and 86% received the full series of doses. Vaccine uptake was lowest among those who were younger, less educated, heavier drinkers and marijuana users, and had fewer comorbidities including lower BMI and diabetes.

**Conclusions:**

While initial vaccination non-intent was high, we observed higher-than-expected and more rapid COVID-19 vaccine uptake among vulnerable women of color engaged in a long-term research initiative relative to Chicago residents overall. Lower education, higher alcohol and marijuana use, and lower COVID morbidity and mortality risks were predictors of not receiving COVID vaccination. Understanding and addressing factors associated with vaccine refusal should be a key component of future pandemic preparedness initiatives.

## Introduction

Coronavirus disease 2019 (COVID-19) spread quickly throughout the world.^1^ In late January 2020, the first confirmed case of COVID-19 was identified in Chicago, Illinois with a rapid increase to over 210,000 confirmed cases by December 2020 and over 4,300 deaths. As of September 2023, there have been >750,000 confirmed cases and >8,000 deaths.^2^ To mitigate and control the rapidly accelerating pandemic, the US government announced “Operation Warp Speed” focused on the development and distribution of vaccines and therapies.^3^ In Chicago, as in much of the United States, Black and Hispanic residents, as well as individuals 40 years or older, have been disproportionately affected by the COVID-19 pandemic. Racial inequities in confirmed Chicago cases and deaths related to COVID-19 are glaringly evident; 40% of the people in Chicago who died in 2020 from the virus were Black, while <30% of Chicagoans are Black.^4^

Vaccine hesitancy, the reluctance or refusal to vaccinate regardless of availability of vaccinations, was a barrier to immunization programs in the United States long before the COVID-19 pandemic.^5^ However, general misinformation, spread widely through the media, and the politicization of the pandemic have emerged as major obstacles to the overall success and uptake of the COVID-19 vaccine. Misinformation combined with deep mistrust driven by historical racial mistreatment and current inequities in our healthcare system has threatened and continue to threaten widespread acceptance of the vaccine.^6^ Early studies aimed at assessing vaccine hesitancy/intent prior to Food and Drug Administration (FDA) authorization, showed that COVID-19 vaccine hesitancy was highest among Black persons when compared to other racial/ethnic groups.^1,6,7^

The women enrolled at the Chicago Cook County site of the Multicenter AIDS Cohort Study (MACS)/Women’s Interagency HIV Study (WIHS) Combined Cohort Study (MWCCS) represent a population that is at high risk of severe illness caused by COVID-19.^8^ In addition, these women live in areas with high community vulnerability indices- an index adapted from the CDC social vulnerability index to predict communities at high risk of severe COVID-19 disease.^9^ These women would particularly benefit from COVID-19 vaccination: the average age is over 50 years, over half are Black, and more than two-thirds are women with HIV (WWH).

With ongoing emergence of viral variants and anticipation of future pandemics, vaccine uptake has remained essential to prevent infection, reduce transmission, and mitigate virus-related complications. Therefore, our primary aim was to learn from our cohort of women who have experienced multiple marginalizing social and health inequities, what factors most influenced their initial COVID-19 vaccine intent, subsequent uptake, and persistent refusal over time.

## Methods

### Study population

Participants were from the Chicago site of the WIHS, now the MWCCS, an ongoing, multicenter longitudinal cohort study of HIV in the United States. Enrollment in the WIHS began in 1994–1995 and was followed by three additional enrollment waves in 2001–2002, 2011–2012, and 2013–2015. The WIHS study protocol and cohort characteristics have been previously described.^10,11^ During the height of the COVID-19 pandemic, in-person cohort study visits were halted and research staff focused on frequent contact with participants, repeatedly providing COVID-19 information detailing safety measures/practices and vaccine preparedness to participants through mailed flyers, telephone calls, and a Zoom town hall. Beginning in April 2020, a series of four monthly telephone interviews were conducted to assess the effect of COVID-19 infection and impact of the pandemic among people with HIV (PWH). During those calls, participants from the Chicago site of the WIHS were provided with additional opportunities for Q&A with research staff.

As part of the fourth monthly phone interview (August−September 2020) Chicago WIHS/MWCCS women were asked questions to assess COVID-19 knowledge, attitudes, and practices (KAP survey), thoughts on re-opening practices, and lived experiences during the pandemic. All initial demographic, behavioral, and clinical data used for this analysis were carried forward from the participant’s most recent semiannual research visit, typically in late 2019 or early 2020, or self-reported during a telephone interview during COVID closures. Follow-up data, including vaccinations and covariates, were from the closest MWCCS visit occurring from October 2020 to September 2023. All study participants provided written informed consent after review and approval by the Cook County Health Institutional Review Board.

### Vaccine intent, uptake, and refusal

As part of the COVID-19 KAP survey, prior to FDA authorization of vaccines, women were asked to respond to the statement “I would choose to be vaccinated once scientists develop and confirm that a vaccine is effective against the virus that causes COVID-19?” Participants could either respond that they felt the statement was “True” or “False.” Those that responded “False,” were considered to report no intent to vaccinate. From March to September 2021, after FDA approval and COVID-19 vaccine availability became more widespread, Chicago WIHS/MWCCS re-enrolled women completed additional questionnaires by mail to ascertain their current COVID vaccination status and related decision-making. Beginning in April 2021, vaccination status and dates of administration were collected locally during resumed in-person visits. Vaccine uptake was determined by receipt of at least one dose of a COVID-19 vaccine. Participants who completed a vaccine series (*i.e.,* two doses of mRNA vaccines or one of Johnson and Johnson’s vaccine) were considered fully vaccinated. Women that remained unvaccinated at the end of data collection, regardless of initial intent, were considered vaccine refusals.

### Covariates

We selected covariates based on knowledge of COVID-19 risk and additional *a priori* factors of interest including HIV serostatus, age, race/ethnicity (Black, Hispanic, other, White), highest level of education, annual household income, employment, housing, cigarette, alcohol, and drug use, drug treatment program, general anxiety disorder (GAD-7, cutoff score of 10), Perceived Stress Scale (PSS-10, cutoff of upper tertile of 18), and Center for Epidemiologic Studies Depression Scale (the shortened CES-D was used with the initial vaccine intent, and the full CES-D, cutoff score of 16, was collected with the follow-up data), body mass index (BMI; kg/m^2^), asthma, chronic obstructive pulmonary disease (COPD), hypertension, diabetes, cancer diagnoses, and prior flu vaccine. The comorbidity burden was determined based on current obesity (BMI ≥30), hypertension, diabetes, and COPD. Racial/ethnicity-based medical mistrust was measured using the 12-item Group-Based Medical Mistrust Scale.^12^ Variables of interest for WWH also included CD4 count and viral load (>20 copies/mL vs. undetectable).

### Statistical methods

Descriptive statistics were used to analyze demographic, behavioral, and clinical characteristics in the overall sample. Differences in cohort characteristics by initial COVID-19 vaccine intent were compared using Chi-square tests or (Fisher’s exact when cells are <5) for categorical variables and Wilcoxon rank-sum (median and IQR) or *t*-tests (mean and SD) for continuous variables. To identify predictors of vaccine intent, odds ratios and 95% confidence intervals were calculated in bivariate analyses. Qualitative data (short narrative responses) on primary reasons for receipt/intent to receive the COVID-19 vaccine were coded into key themes. Frequently reported themes were summarized by vaccination status (receipt of any dose). Vaccine uptake prevalence from November 2020 to September 2023 was also assessed. Predictors of COVID-19 vaccine uptake to assess persistent vaccine refusal were compared using Chi-square tests or (Fisher’s exact when cells are <5) for categorical variables and Wilcoxon rank-sum (median and IQR) or *t*-tests (mean and SD) for continuous variables. All analyses were performed using SAS software, version 9.4 (SAS Institute, Inc. Cary, NC). A *p* value <0.05 was considered statistically significant.

## Results

A total of 146 participants (102 WWH/44 women without HIV [WWoH]) who responded to the vaccine intent question and had known subsequent vaccination status through September 2023 were included. Cohort characteristics at time of the COVID-19 KAP survey administration (August–September 2020) are presented in Table 1. The mean age of participants was 54.4 years (range 36.9–78.8 years). Most participants were Black (63%), had ≤$18,000 annual household income (58%), had a high school education or less (63%), and were unemployed (73%). Among WWH, 17% had detectable viral loads (>20 copies/mL) and 10% had a CD4 count of less than 350 cells/mm^3^. Almost 2/3 had a flu vaccine in the past year (64%). Over a third (40%) of women had been tested for COVID-19 with only 3 (5%) positive cases. Burden of comorbidities (COPD, hypertension, diabetes, and obesity: BMI ≥30) was high among participants, with almost two thirds of women reporting at least one comorbidity (65%) (Table 1).

**Table 1.** Demographics of 146 Chicago MWCCS Women Who Participated in the COVID-19 KAP

Variable	Overall
	*N* (%)
Age, mean (SD)	54.4 (8.5)
Min.–Max.	[36.9–78.8]
<45 years	19 (13.0)
45–54 years	55 (37.7)
55–64 years	59 (40.4)
65+ years	13 (8.9)
Race/ethnicity	
Black	92 (63.0)
Hispanic	21 (14.4)
Other	23 (15.8)
White	10 (6.8)
Average annual household income, ≤$18,000	80 (57.6)
Education	
<High school	51 (34.9)
High school	41 (28.1)
>High school	54 (37.0)
Employed	
Full-time	22 (15.3)
Part-time	17 (11.8)
Unemployed	105 (72.9)
Housing	
Own home	118 (81.9)
Someone else’s home	20 (13.9)
Shelter/homeless	6 (4.2)
High anxiety (GAD-7 ≥ 10)	24 (16.7)
High stress (PSS-10 ≥ 18)	47 (32.4)
High depressive symptoms (CES-D ≥ 10)	28 (19.2)
Alcohol use	
>7 drinks/week	12 (8.4)
≤7 drinks/week	67 (46.8)
Abstainer	64 (44.8)
Marijuana use, yes	37 (25.9)
Multiple illicit drug use^a^, yes	16 (11.2)
Drug treatment program, yes	22 (15.4)
Ever flu vaccine, yes	114 (79.2)
Recent flu season, yes	92 (63.9)
Been tested for COVID-19, yes	58 (39.7)
Positive COVID-19 test, yes	3 (5.2)
HIV, seropositive	102 (69.9)
Detectable viral load, yes	17 (16.7)
CD4 count, <350	10 (9.8)
BMI, mean (SD)	31.7 (8.4)
Underweight: <18.5	2 (1.4)
Normal: 18.5–24.9	27 (19.6)
Overweight: 25.0–29.9	38 (27.5)
Obese: ≥30	71 (51.5)
Cigarette smoker	
Current	69 (48.2)
Former	29 (20.3)
Never	45 (31.5)
Asthma, yes	30 (20.8)
COPD, yes	14 (9.7)
Hypertension, yes	52 (36.1)
Diabetes, yes	20 (13.9)
Ever cancer diagnosis, yes	24 (16.7)
Comorbidity burden^b^	
0 diagnoses	51 (34.9)
1 diagnosis	50 (34.3)
2 diagnoses	28 (19.2)
3 diagnoses	17 (11.6)

Due to missing data, n’s may not add up to total *n* = 146. Data presented as counts (frequencies), unless otherwise noted.

^a^ Crack cocaine, other form of cocaine, speed/meth/ice, or heroin.

^b^ COPD, hypertension, diabetes, and/or obesity (BMI ≥30).

BMI, body mass index; CES-D, Center for Epidemiologic Studies Depression Scale; COPD, chronic obstructive pulmonary disease; COVID-19, coronavirus disease 2019; GAD-7, General Anxiety Disorder-7; HIV, human immunodeficiency virus; KAP, knowledge, attitudes, and practices; MWCCS, MACS/WIHS Combined Cohort Study; PSS-10, Perceived Stress Scale.

Of the 146 participants, 67 (45.9%) said, at the initial August/September 2020 timepoint, that they would not choose to be vaccinated once scientists developed and confirmed that the vaccine is effective against the virus. Compared to those unemployed (40%, 42/105), women who were employed (62%, 24/39) were more likely to report no intent to vaccinate (OR = 2.40, 95% CI: 1.13–5.10, *p* = 0.021). Among comorbidities, WWH (38%) were significantly less likely to report no intent to vaccinate, than WWoH (64%) (OR = 0.35, 95% CI: 0.17–0.74, *p* = 0.005). There were no significant differences in other comorbidity burden by vaccine intent. (Table 2). Women reporting no intent to vaccinate were more likely to agree/strongly agree that people of their racial/ethnic group were treated like “guinea pigs” by doctors and health care workers (*p* = 0.055) (data not shown).

**Table 2.** Sociodemographics, Comorbidities, and COVID-19 Risk Factors of 146 Chicago MWCCS Women by Vaccine Intent

Variable	Intent to be vaccinated			
	No, 67 (45.9)	Yes, 79 (54.1)	*p* Value^a^	Unadjusted or (95% CI)
	*N* (%)	*N* (%)		
Age, mean (SD)	53.6 (9.1)	55.2 (7.9)	0.264	0.98 (0.94–1.02)
<45 years	12 (17.9)	7 (8.9)	0.186	1.07 (0.25–4.59)
45–54 years	24 (35.8)	31 (39.2)		0.48 (0.14–1.67)
55–64 years	23 (34.3)	36 (45.6)		0.40 (0.12–1.37)
65+ years	8 (12.0)	5 (6.3)		Ref.
Race/ethnicity				
Black	44 (65.7)	48 (60.8)	0.424	3.67 (0.74–18.2)
Hispanic	10 (14.9)	11 (13.9)		3.64 (0.62–21.4)
Other	11 (16.4)	12 (15.2)		3.67 (0.64–21.1)
White	2 (3.0)	8 (10.1)		Ref.
Average annual income, ≤$18,000	36 (58.1)	44 (57.1)	0.913	1.04 (0.53–2.04)
Education				
<High school	25 (37.3)	26 (32.9)	0.768	1.12 (0.52–2.40)
High school	17 (25.4)	24 (30.4)		0.82 (0.36–1.87)
>High school	25 (37.3)	29 (36.7)		Ref.
Employed^b^, yes	24 (36.4)	15 (19.2)	**0.021**	**2.40 (1.13–5.10)**
Housing				
Own home	56 (84.8)	62 (79.5)	0.231	Ref.
Someone else’s home	6 (9.1)	14 (17.9)		0.47 (0.17–1.32)
Shelter/homeless	4 (6.1)	2 (2.6)		2.21 (0.39–12.6)
High anxiety (GAD-7 ≥ 10)	9 (13.6)	15 (19.2)	0.369	0.66 (0.27–1.63)
High stress (PSS-10 ≥ 18)	17 (25.4)	30 (38.5)	0.093	0.54 (0.27–1.11)
High depressive symptoms (CES-D ≥ 10)	11 (16.4)	17 (21.5)	0.435	0.72 (0.31–1.66)
Alcohol use				
>7 drinks/week	7 (10.6)	5 (6.5)	0.364	1.40 (0.40–4.88)
≤7 drinks/week	27 (40.9)	40 (51.9)		0.68 (0.34–1.35)
Abstainer	32 (48.5)	32 (41.6)		Ref.
Marijuana use, yes	21 (31.8)	16 (20.8)	0.133	1.78 (0.84–3.79)
Multiple illicit drug use^c^, yes	6 (9.1)	10 (13.0)	0.461	0.67 (0.23–1.95)
Drug treatment program, yes	13 (19.7)	9 (11.7)	0.186	1.85 (0.74–4.66)
Ever flu vaccine, yes	51 (77.3)	63 (80.8)	0.607	0.81 (0.36–1.81)
Been tested for COVID-19, yes	25 (37.3)	33 (41.8)	0.583	0.83 (0.43–1.62)
Positive COVID-19 test, yes	3 (12.0)	0 (0.0)	0.075	—
HIV				
Seropositive	39 (58.2)	63 (79.8)	**0.005**	**0.35 (0.17–0.74)**
Seronegative	28 (41.8)	16 (20.2)		Ref.
Detectable viral load, yes	9 (23.1)	8 (12.7)	0.172	2.06 (0.72–5.90)
CD4 Count, <350	3 (7.7)	7 (11.1)	0.738	0.67 (0.16–2.75)
BMI, mean (SD)	32.2 (8.1)	31.3 (8.7)	0.521	1.01 (0.97–1.06)
Underweight — <18.5	0 (0.0)	2 (2.7)	0.591	—
Normal — 18.5–24.9	13 (20.0)	14 (19.2)		Ref.
Overweight — 25.0–29.9	16 (24.6)	22 (30.1)		0.78 (0.29–2.11)
Obese — ≥30.0	36 (55.4)	35 (48.0)		1.11 (0.46–2.69)
Cigarette smoker				
Current	35 (53.0)	34 (44.1)	0.337	1.18 (0.55–2.50)
Former	10 (15.2)	19 (24.7)		0.60 (0.23–1.58)
No	21 (31.8)	24 (31.2)		Ref.
Asthma, yes	18 (27.3)	12 (15.4)	0.080	2.06 (0.91–4.68)
COPD, yes	5 (7.6)	9 (11.5)	0.424	0.63 (0.20–1.98)
Hypertension, yes	23 (34.9)	29 (37.2)	0.772	0.90 (0.46–1.79)
Diabetes, yes	8 (12.1)	12 (15.4)	0.573	0.76 (0.29–1.98)
Ever cancer diagnosis, yes	10 (15.2)	14 (18.0)	0.654	0.82 (0.34–1.98)
Comorbidity burden^d^				
0 diagnoses	24 (35.8)	27 (34.2)	0.765	Ref.
1 diagnosis	21 (31.3)	29 (36.7)		0.81 (0.37–1.79)
2 diagnoses	15 (22.4)	13 (16.4)		1.30 (0.52–3.27)
3 diagnoses	7 (10.5)	10 (12.7)		0.79 (0.26–2.39)

Bold indicates *p*-value <0.05.

Due to missing data, n’s may not add up to total *n* =146. Data presented as counts (frequencies), unless otherwise noted.

^a^ *p*-value from chi-square tests for categorical variables (Fisher’s exact when cells are <5) or *t*-tests for continuous variables.

^b^ Full-time or part-time employment reported.

^c^ Crack cocaine, other form of cocaine, speed/meth/ice, or heroin.

^d^ COPD, hypertension, diabetes, and/or obesity (BMI ≥30).

BMI, body mass index; CI, confidence intervals; OR, odds ratio.

During August–September 2020 when the COVID-19 KAP survey was collected, there was high COVID-19 symptom knowledge, regardless of intent to vaccinate. The majority of both those who expressed pre-authorization intent versus no intent to be vaccinated could identify correct and incorrect symptoms of a COVID-19 infection (64%–96% accuracy). Among all women, there was low confidence in the US government’s response to COVID, regardless of pre-authorization vaccine intent (76%—did not believe that the US government was doing enough to stop the spread of COVID-19) and most women understood and employed good infection prevention practices such as continuing to wear a mask even after no longer required (88%), maintaining safe social and physical distancing (97%), and continuing to limit social gatherings to 10 people or fewer (90%) (data not shown).

From March to September 2021, 127 (87%) women responded to the self-administered qualitative questionnaires about decision-making regarding their receipt of COVID-19 vaccination and 111 (87%) women stated that they had received or intended to receive the vaccine. The majority of women stated their reasons for intent to vaccinate or vaccine uptake included: protection of self and/or others (54%), health reason/comorbidity risk (21%), and no particular reason (10%). Sixteen (13%) of women had not and did not intend to vaccinate. Their reasons included: safety concerns (44%) and a lack of trust in vaccine/science (31%). Overall, 43 (34%) reported having changed their opinion about initial, preauthorization intention to vaccinate, with the majority changing toward intent to vaccinate. The main reasons for changing their mind about the COVID-19 vaccination included: health reasons/comorbidity risk (14%), fear of death (14%), and protection of themselves and/or others (12%) (Table 3).

**Table 3.** Decision Making regarding COVID-19 Vaccination

	Overall *N* (%)
Main reason for getting vaccinated (*n* = 111)	
Protection of self and/or others	60 (54.1)
Health reasons/comorbidity risk	23 (20.7)
No reason/unknown	11 (9.9)
Get everything back to normal	4 (3.6)
Do their part to fight COVID	3 (2.7)
Encouraged by doctor/government	3 (2.7)
Encouraged by someone close to them	3 (2.7)
Confidence in vaccine	2 (1.8)
Had COVID	1 (0.9)
Incentivized	1 (0.9)
Main reason for not getting vaccinated (*n* = 16)	
Safety concern	7 (43.8)
Lack of trust in vaccine/science	5 (31.2)
Still thinking	2 (12.5)
Religious belief	1 (6.25)
No reason	1 (6.25)
Main reason for changing opinion about vaccination (*n* = 43)	
Health reasons/comorbidities	6 (14.0)
Fear of death	6 (14.0)
Protection of themselves and/or others	5 (11.6)
Fear of vaccine/vaccine concern	4 (9.3)
Seeing people/someone close to them get the vaccine	4 (9.3)
Had COVID	3 (7.0)
Known someone to die of COVID	3 (7.0)
No reason	3 (7.0)
Trusted the vaccine	3 (7.0)
Researched more about the vaccine	2 (4.6)
Set an example/encourage others to receive	2 (4.6)
Heard negative stories	1 (2.3)
Want to go back to normal	1 (2.3)

**FIG. 1.**
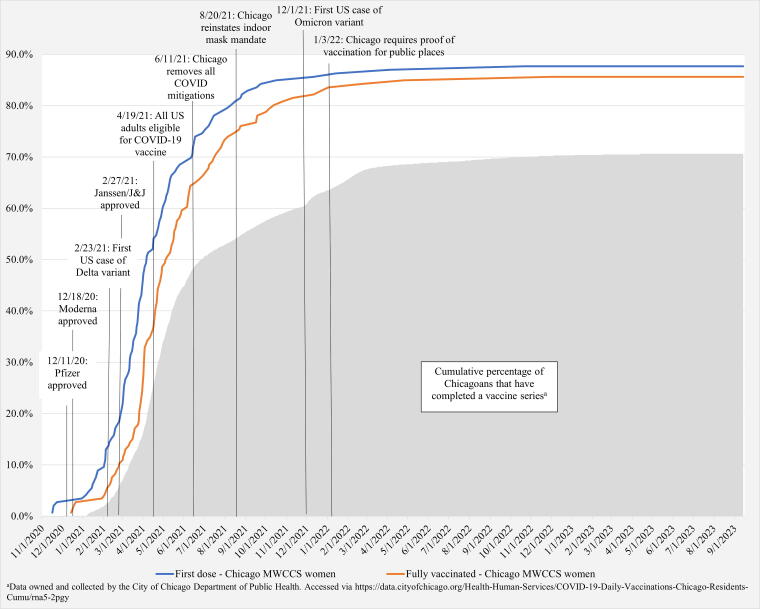
COVID-19 vaccine uptake among Chicago MWCCS women from November 2020 to September 2023. MWCCS, MACS/WIHS Combined Cohort Study.

Of the 67 women who initially reported no intent to vaccinate in the fall preauthorization 2020 survey, 11 (16%) remained persistently vaccine hesitant and had not been vaccinated as of September 2023, whereas seven (9%) of those who stated that they would choose to vaccinate had not yet been vaccinated (*p* = 0.166). There were no significant differences between HIV serostatus groups on current vaccination status. The 18 women not vaccinated by September 2023 were significantly more likely to be younger (*p* = 0.012), have less than a high school education (*p* = 0.0003), heavier drinkers (*p* = 0.024), marijuana users (*p* = 0.049), have a lower BMI (*p* = 0.003), and less likely to have diabetes (*p* = 0.017) compared to those who received a COVID-19 vaccine. (Table 4) More women who had not been vaccinated agreed that people of their racial/ethnic group should be suspicious of modern medicine (47% vs. 14%: *p* = 0.0009). Those not vaccinated were also more likely to believe that they should not confide in doctors or health care workers (29% vs. 7%: *p* = 0.035) and that they have been treated poorly or unfairly by doctors or health care workers (35% vs. 9%: *p* = 0.040). (Data not shown) As of September 2023, 88% of the 146 Chicago MWCCS women had received at least one dose of a COVID-19 vaccine and 86% had been fully vaccinated. Compared to Chicagoans overall, women in this study had a higher rate of completed vaccine series. As of September 2023, 71% of city of Chicagoans had completed a vaccine series (data from the city of Chicago’s COVID-19 data portal)^2^ (Fig. 1).

**Table 4.** Sociodemographics, Comorbidities, and COVID-19 Risk Factors of 146 Chicago MWCCS Women by Vaccine Uptake

Variable	Vaccinated		
	No, 18 (12.3)	Yes, 128 (87.7)	*p* Value^a^
	*N* (%)	*N* (%)	
Age, mean (SD)	51.5 (8.1)	56.9 (8.3)	**0.010**
<45 years	5 (27.8)	8 (6.3)	**0.012**
45–54 years	6 (33.3)	45 (35.2)	
55–64 years	7 (38.9)	51 (39.8)	
65+ years	0 (0.0)	24 (18.7)	
Race/ethnicity			
Black	13 (72.2)	79 (61.7)	0.919
Hispanic	2 (11.1)	19 (14.9)	
Other	2 (11.1)	21 (16.4)	
White	1 (5.6)	9 (7.0)	
Average annual income, ≤$18,000	13 (72.2)	78 (64.5)	0.518
Education			
<High school	11 (61.1)	40 (31.2)	**0.0003**
High school	7 (38.9)	34 (26.6)	
>High school	0 (0.0)	54 (42.2)	
Employed^b^, yes	2 (11.1)	42 (33.3)	0.061
Housing^c^, own home	15 (83.3)	110 (86.6)	0.716
High stress (PSS-10 ≥ 18)	9 (50.0)	41 (34.8)	0.211
High depressive symptoms (CES-D ≥16)	6 (33.3)	37 (31.9)	0.903
Alcohol use			
>7 drinks/week	4 (26.7)	7 (5.9)	**0.024**
≤7 drinks/week	5 (33.3)	67 (56.3)	
Abstainer	6 (40.0)	45 (37.8)	
Marijuana use, yes	8 (44.4)	27 (22.7)	**0.049**
Multiple illicit drug use^d^, yes	4 (23.5)	14 (11.9)	0.244
Ever flu vaccine, yes	14 (77.8)	112 (87.5)	0.274
Been tested for COVID-19, yes	16 (88.9)	119 (93.0)	0.627
Positive COVID-19 test, yes	5 (27.8)	37 (28.9)	0.921
HIV			
Seropositive	12 (66.7)	90 (70.3)	0.752
Seronegative	6 (33.3)	38 (29.7)	
Detectable viral load, yes	5 (45.5)	19 (22.9)	0.107
CD4 count, <350	2 (16.7)	6 (7.0)	0.253
BMI, mean (SD)	29.8 (8.0)	33.1 (9.0)	0.141
Underweight — <18.5	0 (0.0)	1 (0.9)	**0.003**
Normal — 18.5–24.9	9 (50.0)	14 (12.4)	
Overweight — 25.0–29.9	2 (11.1)	35 (31.0)	
Obese — ≥30.0	7 (38.9)	63 (55.7)	
Cigarette smoker			
Current	10 (55.6)	49 (39.2)	0.320
Former	4 (22.2)	48 (38.4)	
No	4 (22.2)	28 (22.4)	
Asthma, yes	6 (33.3)	26 (20.3)	0.211
COPD, yes	1 (5.6)	18 (14.1)	0.469
Hypertension, yes	8 (50.0)	81 (65.9)	0.213
Diabetes, yes	2 (25.0)	35 (71.4)	**0.017**
Comorbidity burden^e^			
0 diagnosis	5 (27.8)	28 (21.9)	0.076
1 diagnosis	8 (44.4)	31 (24.2)	
2 diagnoses	5 (27.8)	45 (35.2)	
3 or more diagnoses	0 (0.0)	24 (18.7)	

Bold indicates *p*-value <0.05.

Due to missing data, n’s may not add up to total *n* = 146. Data presented as counts (frequencies), unless otherwise noted.

^a^ *p* Value from chi-square tests for categorical variables (Fisher’s exact when cells are <5) or *t*-tests for continuous variables.

^b^ Full-time or part-time employment reported.

^c^ Housing: own home vs. any other place.

^d^ Crack cocaine, other form of cocaine, speed/meth/ice, or heroin.

^e^ COPD, hypertension, diabetes, and/or obesity (BMI ≥30).

BMI, body mass index; COPD, chronic obstructive pulmonary disease; SD, standard deviation.

## Discussion

This study demonstrated that prior to the FDA authorization of emergency use of COVID-19 vaccines, initial vaccine intent was low among urban Chicago WWH and demographically similar WWoH enrolled in a long-term observational cohort study. This was evident despite relatively high knowledge of COVID-19 symptoms and prevention practices. Despite a high comorbidity and social vulnerability burden, initial vaccine hesitancy was high, similar to those rates found in other cohorts of PWH at similar timepoints.^13,14^ Initial vaccine intent differed significantly by HIV serostatus and employment: lower among WWoH and unemployed women. We also found prevalent medical mistrust, which was associated with vaccine intent. This study corroborates the high prevalence of vaccine hesitancy prior to vaccine dissemination found in previous studies^13–15^ and expands these findings by following up with participants’ actual vaccination uptake once available.

While initial vaccine intent was low prior to the FDA emergency use authorization, WWoH were much more likely to express no intent to vaccinate (64%) than WWH (38%) (*p* = 0.005). A national survey conducted in the United States among PWH in 2020 showed that 91% of participants had some degree of vaccine hesitancy, much higher than our findings.^14^ However, researchers used multiple items to assess vaccine hesitancy, unlike our single question on vaccine intent and included men and women, unlike our women only study.^14^ It is possible that if we had considered not only vaccine intent but also reasons for hesitancy, such as side effects, safety, and trust, our women would have reported similar levels of vaccine hesitancy. However, the women in this study have been highly engaged, some since 1994, in the larger cohort study which routinely includes health education initiatives such as distributing COVID-19 information throughout the pandemic, which may account for our initial higher rates of vaccine intent.

It is also plausible that the differences seen in HIV serostatus reflect that WWH in our study represent a population that has more opportunities to be proactive in their own health and better engaged in the health care system compared to their counterparts without HIV. Over 30+ years, women enrolled in the WIHS, now MWCCS, have been actively encouraged and provided with referrals to maintain and engage in their health care. They might have already been participating in conversations around future COVID-19 vaccinations with their health care providers and been educated on their risks as WWH. WWoH in this study are generally less involved in managing their own health care, partially due to barriers in access, so it is possible that they just did not have the opportunity to discuss the risk and benefits of vaccination once available. Research on hesitancy for future vaccinations including COVID boosters in this population is warranted to further elucidate the differences related to HIV serostatus and the determinants of refusal to vaccinate.

Our study found similarly high levels of medical mistrust as previous studies, regardless of vaccination intent or uptake.^13,14,16^ Almost a third of the women felt that people of their racial/ethnic group were not treated the same as people of other groups by doctors, while a fifth believed that people of their racial/ethnic group should be suspicious of modern medicine. In a cohort of Black Americans living with HIV, predominantly men, Bogart et al. found that more than half of participants had at least one vaccine or treatment hesitancy belief and medical mistrust around COVID-19 was prevalent.^13^ Mistrust is often rooted in systemic and historical racism and is perpetuated by poverty and racial inequities during health care encounters. Given that almost two-thirds of our participants were Black (63%) women, a high prevalence of medical mistrust is unsurprising. There is a deep mistrust of the health care system with foundations in historical evidence and awareness of systemic racism and discrimination against women.^17^ The recent social and political climate in the United States has further perpetuated feelings of mistrust, which was highly evident in Chicago, particularly during the concurrence of COVID-19 and community policing demonstrations. Public health policy-makers and individual providers should acknowledge this mistrust based on historical transgressions, and use education, engagement, and active listening to repair and build trust.^18^

We did not find a significant difference among ethnoracial groups in regards to vaccine intent in contrast to some previous studies.^13–15^ Chicago is a large metropolitan area with high levels of residential segregation based on race and income. The racial disparities in mortality from the COVID-19 pandemic mirrored that of existing racial inequities in mortality from non-COVID-19 related causes.^19^ A 2020 study conducted in Chicago, found that higher COVID-19 mortality was seen in neighborhoods with greater obstacles to social distancing and lower health insurance coverage. They also found neighborhoods with higher percentages of Black and Hispanic residents had greater COVID-19 mortality rates.^19^ As a result of these evident inequities, the Protect Chicago Plus program was developed to provide consistent, convenient, and equitable access to vaccines for Chicagoans in low vaccinated areas through targeted static and mobile vaccination sites, and an at-home vaccination program.^20^ Through this initiative, it is possible that many women in our study were reached and the previous barriers to intent to vaccinate, whether physical or misinformation/mistrust, were overcome.

In our follow-up conducted after FDA approval, there were no significant differences in vaccination uptake by HIV serostatus. Factors significantly associated with COVID-19 vaccination refusal included younger age, less than a high school education, heavier alcohol use, marijuana use, and lower BMI. These findings suggest that vaccine uptake in this cohort of WWH and WWoH may be driven primarily by greater perceived risk of severe illness from COVID infection among older women.

In a study conducted among 496 PWH in early 2021, Jaiswal et al. found that 64% of participants had received at least one dose of a COVID-19 vaccine. Like our study, they found vaccine uptake to be associated with older age and higher educational attainment. Although they conducted their study of vaccine uptake in a shorter time period than ours (March — May 2021), they also found that at the time of study, the rate of uptake among their participants was higher than the national average.^21^ We found that overall, the majority of our sample (86%) had been fully vaccinated, which was much higher than the prevalence of fully vaccinated city of Chicagoans (71%) as of September 2023.

Although a large subset of participants initially expressed no intent to receive COVID-19 vaccination once available and medical mistrust was pervasive in our sample, the majority of women did in fact get vaccinated once the vaccine was approved. It is possible that greater perceived COVID-19 vulnerability was motivation enough to vaccinate, since the overall rate of vaccination (86%) was high for our sample in relation to national and local averages. Alternatively, it could be that the public health interventions targeting those at high risk successfully overcame hesitancy. Younger women and those with fewer co-morbidities were less likely to eventually get the vaccine. Perhaps the initial prioritization of older adults and those with comorbidities inadvertently downplayed the necessity and urgency of COVID-19 vaccination for younger women and those with fewer health concerns. Qualitative studies to understand specific reasons for persistent vaccine resistance are necessary to create community-based public health messaging for future health crises to successfully reach healthier populations still at risk for acquiring and transmitting infections.

It is also possible that our initiative of an early, proactive, and engaged approach to understanding and addressing vaccine preparedness in our cohort ultimately contributed to high rates of vaccine uptake despite the documented medical mistrust and initial high rate of vaccine hesitancy. If clinical practices and public health systems utilized a low cost, but respectful approach, similar to that used with our study cohort, clinicians, through open dialogue, might reduce mistrust and help patients move toward eventual vaccine acceptance. Our experience during the COVID-19 pandemic has taught us about how to successfully connect with individuals who have a high level of mistrust in the health care system and provides us with lessons and tools to prepare for future epidemics.

### Strengths and limitations

Given the longitudinal approach of our study, we were able to assess vaccine intent and uptake at various timepoints of the COVID-19 pandemic, including prior to and after the FDA authorization of emergency use of vaccines. Also, our sample is unique in that it includes a well-matched group of WWoH and availability of a large number of relevant covariates.

This study had several limitations. The sample included only Chicago-based WWH and WWoH infection. Therefore, the study results may not be generalizable to a broader population or representative of WWH living in other areas of the United States. Our sample is also unique and potentially not representative of the general PWH population as MWCCS participants are frequently engaged in their own care and provided with health information at multiple study visits throughout the year. The use of self-reported data has its inherent limitations as well, including social desirability and recall biases. Also, by not incorporating information about previous experiences with vaccinations including side effects, we could have missed possible personal reasons for vaccine resistance.

## Conclusion

Although COVID-19 vaccine hesitancy was highly prevalent prior to authorization of emergency use of vaccines by the FDA in a cohort of WWH and WWoH, high acceptance and rapid uptake of vaccination were achieved. Our findings suggest that, while not due to a lack of knowledge or misperceptions, the foundations of initial vaccine hesitancy were rooted in mistrust in the health care system driven by historical and systemic racial mistreatment and inequities. Policy makers and providers should be aware of the high degree of medical mistrust in this population and develop approaches to improve communications of public health messages. While our findings also provide important insight into the small subset of women perpetually vaccine resistant, they also provide encouragement that a relatively low cost, proactive approach to engaging participants in understanding and encouraging vaccine preparedness, could ultimately lead to vaccine acceptance.

## Abbreviations Used

AIDS: Acquired immunodeficiency syndrome
BMI: Body mass index
CES-D-10: Center for Epidemiologic Studies Depression Scale
COPD: Chronic obstructive pulmonary disease
COVID-19: Coronavirus disease 2019
FDA: Food and Drug Administration
GAD-7: General Anxiety Disorder-7
HIV: Human immunodeficiency virus
IQR: Interquartile range
KAP: Knowledge, attitudes, and practices
MACS: Multicenter AIDS Cohort Study
MWCCS: MACS/WIHS Combined Cohort Study
OR: Odds ratio
PWH: People with HIV
PSS-10: Perceived Stress Scale
SD: Standard deviation
WIHS: Women’s Interagency HIV Study
WWH: Women with HIV
